# Chemopreventive effect of *Copaifera langsdorffii* leaves hydroalcoholic extract on 1,2-dimethylhydrazine-induced DNA damage and preneoplastic lesions in rat colon

**DOI:** 10.1186/1472-6882-13-3

**Published:** 2013-01-07

**Authors:** Juliana Marques Senedese, Jacqueline Morais Alves, Ildercílio Mota de Souza Lima, Erick Augusto Pedroso de Andrade, Ricardo Andrade Furtado, Jairo Kenupp Bastos, Denise Crispim Tavares

**Affiliations:** 1 Avenida Dr. Armando Salles de Oliveira, Universidade de Franca, 201 Parque Universitário, Franca, São Paulo 14404-600, Brazil; 2 Faculdade de Ciências Farmacêuticas de Ribeirão Preto, Universidade de São Paulo, Avenida do Café S/N, Ribeirão Preto, São Paulo, 14040-903, Brazil

**Keywords:** *Copaifera langsdorffii*, Comet assay, Aberrant crypt foci.

## Abstract

**Background:**

Natural antioxidants present in common foods and beverages have drawn great attention to cancer prevention due to its health benefits, remarkable lack of toxicity and side effects. *Copaifera langsdorffii*, known as “copaiba”, “capaiva”, or “pau-de-óleo“, belongs to the *Leguminosae* family and occurs in fields and grasslands in the northern and northeastern parts of Brazil. Biological studies of *Copaifera* corroborate its widespread use by the population. This paper describes the effects of *C. langsdorffii* leaves hydroalcoholic extract on the 1,2-dimethylhydrazine (DMH)-induced DNA damage and aberrant crypt foci (ACF) in the colon of male Wistar rats.

**Methods:**

The hydroalcoholic extract of *C. langsdorffii* was administered to rats by gavage at daily doses of 20, 40 and 80 mg/kg body weight. To evaluate DNA damage by the comet assay, animals received the *C. langsdorffii* extract for seven days and a single subcutaneous injection (sc) of 1,2-dimethylhydrazine (DMH) at a dose of 40 mg/kg on day 7. Animals were sacrificed 4 h after injection of DMH, to assess DNA damage. For the ACF assay, animals were acclimatized for one week (week 1) and then treated with the *C. langsdorffii* extract five times a week for four weeks (weeks 2 to 5). The rats received sc injections of DMH (40 mg/kg) on days 2 and 5 of weeks 2 and 3, to induce ACF. Animals were euthanized at week 5; i.e., four weeks after the first DMH treatment.

**Results:**

Animals treated with different doses of the *C. langsdorffii* extract combined with DMH had significantly lower frequency of DNA damage as compared with the positive control (animals treated with DMH only). The percentage of reduction in the frequency of DNA damage ranged from 14.30% to 38.8%. The groups treated with 40 and 80 mg/kg *C. langsdorffii* extract during and after DMH treatment presented significantly lower numbers of ACF and aberrant crypts compared with the control.

**Conclusion:**

The *C. langsdorffii* extract significantly reduced the extent of DNA damage and ACF induced by DMH, suggesting that the extract has a protective effect against colon carcinogenesis.

## Background

Colorectal cancer is a major cause of death; its incidence is increasing worldwide [1]. Genetic susceptibility and diet determine cancer risk and tumor behavior [2]. Experimental studies have suggested that plant food components can suppress cancer development through a variety of different mechanisms [3]. Chemoprevention is defined by the use of natural, synthetic, biological, or chemical agents that can reverse, suppress, or prevent carcinogenesis [4].

Plants produce a conspicuous structural diversity of metabolites and represent the largest source of active compounds; they are perhaps the earliest source of drugs for human use [5]. *Copaifera langsdorffii*, popularly known as “copaiba”, “copaíva”, or “pau-de-óleo”, belongs to the family Leguminosae and occurs in fields and grasslands in northern and northeastern Brazil. The *C. langsdorffii* oil-resin is a folk remedy in its natural form and presents several biological activities such as antiparasitic action against *Leishmania amazonensi*s [6] as well as antinociceptive [7], anti-inflammatory [8], analgesic [8], diuretic [9,10], antitumoral [11], antiulcerogenic [12], anti-lipoperoxidation [13], and antioxidant [13] effects.

In this paper we report the chemopreventive potential of the hydroalcoholic extract of *C. langsdorffii* leaves in the colon of male Wistar rats. Researchers have developed several experimental animal models of colon carcinogenesis, to screen chemopreventive agents against colon cancer. Colon carcinogenesis induced by 1,2-dimethylhydrazine (DMH) is histologically, morphologically, and anatomically similar to human colonic epithelial neoplasms [14]. Preneoplastic lesions of the colonic mucosa, the aberrant crypt foci (ACF), are one of the early morphological changes on the DMH-stimulated colonic mucosal surface in rodents [15].

Since it has been indicated that the induction of ACF is clearly related to genotoxic events, primary DNA damage in colon cells is an important end-point for the chemoprevention of colon carcinogenesis. In this context, the comet assay has been used as a rapid and sensitive tool for detecting primary DNA damage in individual cells. The association of chemical-induced comet and ACF assays can provide information on the mechanisms of action of a candidate drug and help determine in which step of carcinogenesis it acts [16].

## Methods

### Plant material and its extraction

Leaves of *Copaifera langsdorffii* Desff were collected in Ribeirão Preto, São Paulo, Brazil and identified by Dr. Milton Groppo Junior from Faculdade de Filosofia, Ciências e Letras de Ribeirão Preto, University of São Paulo, Ribeirão Preto, São Paulo, Brazil; a voucher specimen (SPFR 10120) was deposited in the herbarium of the aforementioned institution. Leaves were dried at 40°C with air circulation and grounded in a knife mill. The crude hydroalcoholic extract was obtained by macerating 200 g of the leaves powder in ethanol/water 7:3 three times, every 48 h. The filtered extracts were combined, concentrated under vacuum, and lyophilized, which furnished 38 g of the extract.

### HPLC analysis of the crude hydroalcoholic extract of *Copaifera langsdorffii* leaves

HPLC analyses were conducted on an HPLC Shimadzu SCL-10Avp (Kyoto, Japan) multisolvent delivery system equipped with a Shimadzu SPD-M10Avp photodiode array detector and an Intel Celeron computer; two monolithic columns linked in series (Onyx™ 100 X 4.6 mm – C_18_ Phenomenex) and a pre-column from the same company were also used. The mobile phase consisted of water and acetonitrile. The elution program was 5 – 6% of acetonitrile in water (v/v) in the first minute, followed by 6 – 8% (1–2 min.), 8 – 10% (2 – 5 min.), 10 – 15% (5 – 12 min.), 15% (12 – 22 min.), 15 – 25% (22 – 27 min.), 25% (27 – 35 min.), 25 – 40% (35 – 39 min.), 40% (39–42 min.), 40 – 100% (42–47 min.), and 100% acetonitrile for another minute, at a flow rate of 1.0 mL min^-1^; twelve additional minutes were allowed for the column to return to the initial conditions and re-equilibrate. The chromatogram peaks were detected at 257 nm. HPLC-grade solvents were purchased from Tedia Company INC. (Fairfield, OH, USA). Water was purified using the Milli-Q-plus filter system (Millipore, Bedford, MA, USA.). Quercetin-3-O-*α*-L-rhamnopyranoside (quercitrin) and kaempferol-3-O-*α*-L-rhamnopyranoside (K-rham) had been previously isolated in our laboratory.

### Animals

Male Wistar rats aged five weeks and weighing approximately 120 g were obtained from the animal house of the Faculty of Pharmaceutical Sciences of Ribeirão Preto, University of São Paulo (Ribeirão Preto, São Paulo, Brazil) and acclimatized for a period of one week before the beginning of the experiments (week 1). Animals were maintained in a room under controlled conditions of temperature (22 ± 2°C), humidity (50 ± 10%), and a 12-h light/dark cycle; standard rat chow and water were available *ad libitum*. The study protocol was approved by the Ethics Committee for Animal Care of the University of Franca (process nº 014/11).

### Carcinogen treatments

The well-known colon carcinogen 1,2-dimethylhydrazine (DMH, Sigma-Aldrich) was dissolved immediately before use in 1 mM EDTA (ethylenediamine tetraacetic acid). For the comet assay, a single DMH dose of 40 mg/kg (kg corresponds to the animal body weight throughout this work) was administered to the animals. For the ACF assay, a total DMH dose of 160 mg/kg divided into four subcutaneous (sc) injections of 40 mg/kg was administered twice a week for two weeks (weeks 2 and 3), as described by Furtado et al. [17].

### Comet assay

Groups of six animals were distributed as follows: negative control (EDTA, 0.05 mL/10 g, sc); positive control (DMH, 40 mg/kg); solvent control (dimethylsulfoxide, DMSO, Sigma-Aldrich, 0.6 g/kg); treated with the *C. langsdorffii* extract (80 mg/kg); treated with the solvent solution plus DMH; and treated with the *C. langsdorffii* extract (20, 40, and 80 mg/kg) plus DMH. The *C. langsdorffii* doses were selected on the basis of literature data [18]. The extract was dissolved in 14% DMSO in water; the treatment groups received similar DMSO doses. Animals were treated with the *C. langsdorffii* extract by gavage (1 mL per animal) for seven consecutive days, and injected with 40 mg/kg DMH on day 7. Body weight and water consumption were measured on a daily basis throughout the experimental period. Animals were anesthetized with sodium pentobarbital (45 mg/kg; i.p.) and euthanized on day 7, 4 h after treatment with DMH or EDTA. After laparotomy, the colon was excised, tied at one extremity, and flushed with saline, to remove feces. The other extremity was tied, too, and an enzymatic cocktail (0.3 mg collagenase I + 5 mg trypsin/EDTA) was injected into the colon. Next, the colon containing the enzymatic cocktail was placed into phosphate-buffered saline (PBS) and kept in a water bath at 37°C for 40 min; one colon extremity was cut off to collect the cell suspension. Cell viability was determined on portions of the cell suspension using a dual-dye assay based on a combination of acridine orange and ethidium bromide (under a fluorescent microscope, viable cells that metabolize acridine orange appear green). To accomplish this assay, a 20 μL aliquot of the dye solution was mixed with 20 μL of the cell suspension. Two hundred cells were counted per animal; cells with viability ≥ 80% were used for the comet assay.

**Figure 1:**
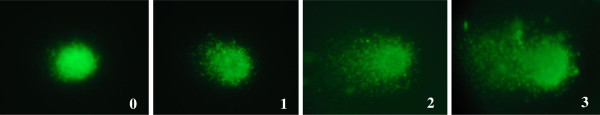
**Images of the comets observed for the colon cells of Wistar rats.** They represent classes 0–3 as used for visual scoring (magnification: 1000 X; SYBR gold).

The alkaline comet assay was performed according to Singh et al. [19] and Burlinson et al. [20]. Briefly, 20 μL of the colon cell suspension were mixed with 120 μL of a 0.5% low-melting-point agarose and layered on a slide precoated with a thin layer of normal-melting-point agarose. The slides were placed into a lysis solution (2.5 M NaCl, 100 mM EDTA, 10 mM Tris, 1% sodium laurylsarcosine, pH 10; with 1% Triton X-100 and 10% DMSO added just before use) for 24 h. The slides were washed in PBS and placed into a horizontal electrophoresis unit filled with freshly prepared alkaline buffer (1 mM EDTA and 300 mM NaOH, pH 13). After 20 min of DNA unwinding, electrophoresis was carried out in the same buffer at 25 V and 300 mA for 20 min. The slides were neutralized (0.4 M Tris, pH 7.5), fixed with 100% ethanol, stained with 40 μL of SYBR gold (10,000X concentrate in DMSO; 1 μL/500 μL distilled water), and covered with a coverslip. The stained comets were immediately evaluated at 1000 X magnification under a Nikon fluorescence microscope fitted with a 515–560 nm excitation filter and a 590 nm barrier filter. For each treatment, the extent and distribution of DNA damage indicated by the comet assay were evaluated by examining 100 randomly selected and non-overlapping comets on the slides (i.e., 600 comets per treatment). For each slide, the comets were visually scored and allocated to one of four classes (0, 1, 2, and 3) according to tail size, as follows: class 0 − undamaged and no tail, class 1− a short tail with length smaller than the diameter of the head (nucleus), class 2 − tail length between one and two times the diameter of the head, and class 3 − maximal damage: a long tail measuring more than twice the diameter of the head (Figure 1). The few comets containing no head and those with almost all DNA in the tail, or with a very wide tail, were excluded from the analysis since they may correspond to dead cells [21]. The total score for 600 comets was calculated according to the modified formula of Manoharan and Banejee [22], as shown below:

$$\text{score}=\left(1\;x\;n_{1}\right)+\left(2\;x\;n_{2}\right)+\left(3\;x\;n_{3}\right),$$

where *n* is the number of comets in each analyzed class. Thus, the total score ranged from 0 to 600.

The percent reduction in DMH-induced damage elicited by the *C. langsdorffii* extract was calculated according to Waters et al. [23] using the following formula:

$$\%\text{Re}\text{duction}=\frac{A-B}{A-C}\;x\;100,$$

where *A*, *B*, and *C* correspond to the mean score obtained for the group treated with DMH (positive control), the *C. langsdorffii* extract plus DMH, and EDTA (negative control), respectively.

### Aberrant crypt foci assay

**Figure 2:**
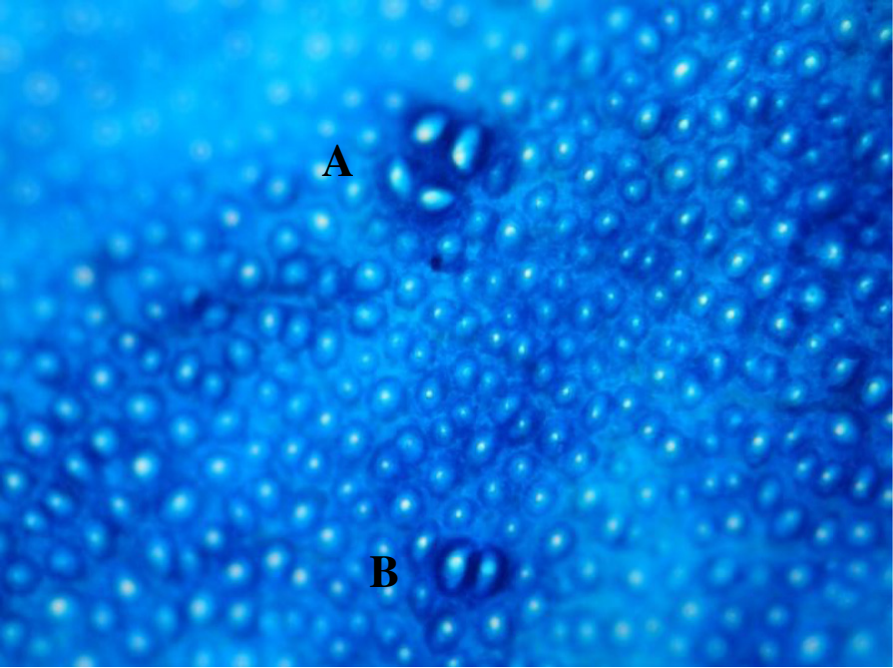
Photomicrograph of methylene blue-stained, whole-mount preparations of colon of Wistar rats treated with DMH with ACF containing four (A) and two (B) aberrant crypts/focus.

Each treatment group consisted of six animals fed with standard chow throughout the experiment (5 weeks). After being acclimatized for one week (week 1), the animals were divided into eight treatment groups: animals treated with 80 mg/kg *C. langsdorffii* extract; DMH (160 mg/kg, positive control); solvent (DMSO; 0.6 g/kg) plus DMH; *C. langsdorffii* extract (at 20, 40, and 80 mg/kg) plus DMH; EDTA (negative control); and DMSO (solvent control). The study period of four weeks was selected on the basis of previous works, which considered four weeks enough to observe ACF formation of [24,25]. The negative and positive controls received EDTA (0.05 mL/10 g) or DMH (40 mg/kg), respectively, twice a week for two weeks (weeks 2 and 3). DMH and EDTA were administered by subcutaneous injections. The *C. langsdorffii* extract and solvent solution were administered to rats five times a week for four weeks (weeks 2 to 5) by gavage (1 mL per animal), during and after DMH treatment. Body weight and water consumption were measured five times a week throughout the experimental period. All the animals were anesthetized with sodium pentobarbital (45 mg/kg; i.p.) and euthanized at week 5 (four weeks after the first DMH administration). After laparotomy, the distal colon was excised, flushed with 0.9% saline, cut open along the longitudinal axis, and fixed in 10% phosphate-buffered formalin (pH 6.9-7.1) for 24 h. Immediately before analysis, the colon was stained with 0.02% methylene blue for 5 min, mounted on microscope slides with the mucosal side facing upward, and observed under a light microscope at 100x magnification. Fifty sequential fields of the distal colon were screened for ACF, which were characterized by elongated, slit-shaped lumens surrounded by thickened epithelium that stained more intensely than the surrounding normal crypts [26]. According to Bird and Good [27], aberrant crypt (AC) initially appears in isolation; after some time, ACF with an additional crypt can be observed (Figure 2). The new crypts generally derive from previously formed AC. Thus, the number of crypts per ACF, called the "multiplicity of crypts", is an important parameter to evaluate ACF progression [28]. Each colon specimen was examined by at least three observers in a double-blind manner.

### Statistical analysis

All the data were analyzed statistically by analysis of variance for completely randomized experiments, with calculation of the F statistics and respective p values. In cases in which *P* < 0.05, treatment means were compared by the Tukey test, and the minimum significant difference was calculated for α = 0.05.

## Results

**Figure 3:**
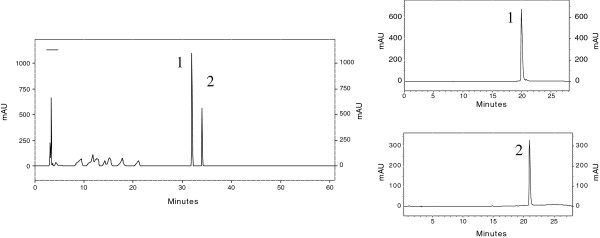
**A, B and C Chromatographic profile of the hydroalcoholic extract of the leaves of *C. langsdorffii *at 257 nm (A); Chromatogram of the standard quercetin-3-O-*α*-L-rhamnopyranoside 1 (B); Chromatogram of the standard kaempferol-3-O-*α*-L-rhamnopyranoside 2 (C).**

The HPLC analysis of the hydroalcoholic extract of *C. langsdorffii* leaves allowed us to quantify the flavonols quercetin-3-O-*α*-L-rhamnopyranoside 1 and kaempferol-3-O-*α*-L-rhamnopyranoside 2 as major compounds (Figure 3).

Regarding the biological assays, Tables 1 and 2 contain the means of the final body weight, body weight gain, and water consumption of the animals during the experimental period. The groups did not differ in terms of these variables.

**Table 1:** **Final body weight, body weight gain, and water consumption of rats treated with different doses of the *C. langsdorffii *extract and/or DMH and their respective controls, for the comet assay**

**Treatments (mg/kg)**	Final body weight (g)*	Body weight gain (g)*	Water consumption*^a^
EDTA	166.8 ± 11.6	54.5 ± 7.9	36.9 ± 5.6
DMSO	170.6 ± 11.0	51.1 ± 6.3	41.5 ± 4.7
80	167.5 ± 11.0	54.6 ± 5.8	40.6 ± 7.4
DMH	165.0 ± 11.3	52.6 ± 7.4	37.2 ± 6.5
DMSO + DMH	168.1 ± 10.4	56.6 ± 7.6	44.6 ± 12.3
20 + DMH	167.1 ± 11.4	52.3 ± 9.3	37.9 ± 5.8
40 + DMH	168.1 ± 11.3	49.3 ± 6.3	37.5 ± 6.8
80 + DMH	169.6 ± 10.2	53.3 ± 6.9	45.5 ± 7.2

*EDTA*, vehicle control; *DMH*, 1,2-dimethylhydrazine (40 mg/kg); *DMSO*, dimethylsulfoxide (0.6 g/kg). The numbers 20, 40, and 80 correspond to the extract dose in mg/kg.

*Values are the mean ± standard deviation.

^a^Water consumption calculated in mL per animal per day.

**Table 2:** **Final body weight, body weight gain, and water consumption of rats treated with different doses of the *C. langsdorffii *extract and/or DMH and their respective controls, for the aberrant crypt foci (ACF) assay**

**Treatments (mg/kg)**	Final body weight (g)*	Body weight gain (g)*	Water consumption*^a^
EDTA	326.8 ± 12.5	215.6 ± 11.9	37.5 ± 6.8
DMSO	335.6 ± 11.2	214.5 ± 11.7	37.5 ± 4.4
80	325.5 ± 12.8	219.0 ± 11.5	37.5 ± 6.8
DMH	321.8 ± 10.1	222.5 ± 10.7	37.9 ± 5.8
DMSO + DMH	334.3 ± 11.3	223.5 ± 11.0	45.5 ± 7.2
20 + DMH	334.5 ± 10.9	224.3 ± 10.8	37.2 ± 6.5
40 + DMH	341.8 ± 11.5	221.8 ± 11.1	44.6 ± 12.3
80 + DMH	340.5 ± 12.1	225.5 ± 11.8	55.0 ± 18.7

*EDTA*, vehicle control; *DMH*, 1,2-dimethylhydrazine (160 mg/kg); *DMSO*, dimethylsulfoxide (0.6 g/kg). The numbers 20, 40, and 80 correspond to the extract dose in mg/kg.

*Values are the mean ± standard deviation.

^a^Water consumption calculated in mL per animal per day.

The comet assay did not reveal any significant differences between animals treated with the *C. langsdorffii* extract only (80 mg/kg) and the negative control group (receiving EDTA only, *P* > 0.05), showing that the extract does not display genotoxicity (Table 3). The extent of DNA damage was significantly higher for the group receiving DMH only (positive control) as compared with the negative control. Animals treated with different doses of the *C. langsdorffii* extract combined with DMH had significantly less DNA damage as compared with the positive control − DNA damage decreased between 14.3% and 38.8%, with the *C. langsdorffii* extract exerting a non-significant dose-dependent protective effect. Animals treated with the solvent solution plus DMH and the positive control did not differ with respect to DNA damage. Cell viability was higher than 95% for all the treatment groups.

**Table 3:** **DNA migration in the comet assay observed in Wistar rat colon treated with different doses of the *C. langsdorffii extract *and/or DMH and their respective controls**

**Treatments (mg/kg)**	Comet class*				*Score	**Reduction**
	***0***	***1***	***2***	***3***		**(%)**
Control	66.2 ± 4.3	23.2 ± 5.7	7.3 ± 2.3	3.3 ± 1.8	47.8 ± 6.4	-
DMSO	65.3 ± 3.9	21.2 ± 4.4	8.3 ± 2.4	3.7 ± 2.2	48.8 ± 4.8	-
80	79.7 ± 5.1	14.7 ± 6.3	5.0 ± 2.6	0.7 ± 0.8	26.7 ± 6.0	-
DMH	13.2 ± 2.1	26.2 ± 5.3	33.5 ± 5.2	27.3 ± 3.7	175.2 ± 9.6^a^	-
DMSO + DMH	11.5 ± 1.6	23.8 ± 9.5	32.8 ± 8.3	28.7 ± 4.3	175.5 ± 11.7^a^	-
20 + DMH	10.8 ± 4.5	40.2 ± 3.6	25.0 ± 6.4	22.3 ± 4.3	157.2 ± 12.2^a,b^	14.3
40 + DMH	19.3 ± 6.3	34.0 ± 4.3	29.7 ± 3.6	16.8 ± 5.9	143.8 ± 15.1^a,b^	24.7
80 + DMH	23.3 ± 6.9	40.8 ± 7.1	20.8 ± 3.5	15.0 ± 5.9	125.8 ± 12.7^a,b,c^	38.8

A total of 600 cells were examined per treatment; *DMH*, 1,2-dimethylhydrazine (40 mg/kg). The numbers 20, 40, and 80 correspond to the extract dose in mg/kg.

*Values are the mean ± standard deviation.

^a^Significantly different from the control (*P* < 0.05).

^b^Significantly different from the DMH group (*P* <0.05).

^c^Significantly different from the 20 + DMH group (*P* < 0.05).

No ACF were observed in the negative control and in the groups receiving *C. langsdorffii* extract (80 mg/kg) or DMSO (data not shown), but only in DMH-treated rats. The number of ACF and the number of AC were significantly lower in the groups treated with 40 and 80 mg/kg *C. langsdorffii* extract during and after DMH treatment as compared with the group treated with DMH only (Table 4). The results for the animals treated with DMSO plus DMH did not differ significantly from those obtained for the animals treated with DMH. The number of AC and the AC/ACF ratio obtained for all the groups treated with DMH revealed higher frequency of foci with one crypt.

**Table 4:** **Mean number (± SD) of aberrant crypt foci (ACF) and aberrant crypt (AC) observed in the distal colon of rats treated with the *C. langsdorffii *extract and DMH**

**Treatments (mg/kg)**	**Number of ACF**				**Number of ACF**	**Number of AC**	**AC/ACF**
	***1***	***2***	***3***	***4***			
DMH	9.2 ± 1.2	3.2 ± 1.3	1.2 ± 0.9	0.5 ± 0.8	14.0 ± 1.4	20.8 ± 1.9	1.5 ± 0.1
DMSO + DMH	6.7 ± 2.6	3.5 ± 1.9	2.2 ± 1.5	0.3 ± 0.8	12.8 ± 3.5	21.0 ± 5.9	1.6 ± 0.2
20 + DMH	7.2 ± 1.6	4.8 ± 1.7	2.2 ± 0.8	0.2 ± 0.4	14.3 ± 2.7^b^	23.7 ± 5.3^b^	1.6 ± 0.2
40 + DMH	6.2 ± 2.3	4.0 ± 0.7	0	0.4 ± 0.5	10.6 ± 1.9^a^	15.8 ± 2.9^a^	1.5 ± 0.2
80 + DMH	4.7 ± 0.8	2.7 ± 1.2	0.7 ± 0.8	0.2 ± 0.4	8.0 ± 1.9^a^	12.0 ± 4.0^a^	1.5 ± 0.2

*DMH*, 1,2-dimethylhydrazine (160 mg/kg); *DMSO*, dimethylsulfoxide (0.6 g/kg). The numbers 20, 40, and 80 correspond to the extract dose in g/kg.

*Values are the mean ± standard deviation.

^a^Significantly different from the DMH group (*P* < 0.05).

^b^Significantly different from the 40 + DMH and 80 + DMH groups (*P* < 0.05).

## Discussion

Because colorectal cancer is an important cause of death in many countries, we evaluated the effect of the hydroalcoholic extract of *C. langsdorffii* leaves on this neoplasia. The sequence of events that culminates in colon cancer helped us select the bioassays and protocols for this study. We employed the two endpoints − chemical-induced DNA damage and ACF − because they help determine in which step of colon carcinogenesis the *C. langsdorffii* extract acts.

ACF are putative preneoplastic lesions of colonic neoplasia in rodents and humans [29]; they are useful intermediate biomarkers that help one to assess the modifying effects of certain natural and synthetic compounds on chemically induced carcinogenesis [30]. The advantage of studying the pathogenesis of cancer of the colon in animal models induced by chemical carcinogens, is that the tumor induction is rapid and allows to reproduce the adenoma-carcinoma sequence, as in humans. The high frequency of tumors that develop in the distal colon of rats, and the histogenesis of multiple adenomas, with consequent formation of adenocarcinomas, justify the importance of this species in the study of the pathogenesis of colon cancer [31].

Treatment with the *C. langsdorffii* extract reduced DNA damage and the number of ACF in the colon tissue of DMH-treated rats. ACF appear to arise from gene mutations, so an increased number of ACF reflects the initiation step of colorectal carcinogenesis. Our results indicate that the *C. langsdorffii* extract prevents the DNA damage and formation of preneoplastic lesions involved in the initial phase of colon cancer. In a previous study, treatments with *C. langsdorffii* extract diminished the genotoxicity induced by the chemotherapeutic agent doxorubicin as revealed by the Swiss mice peripheral blood micronucleus test [18].

*In vivo* studies have shown that DMH is metabolized to azomethane, azoxymethane, methylazoxymethanol, ethane, and carbon dioxide [32]. Furthermore, DMH has been reported to induce carcinogenesis in rats and mice due to the high production of reactive free radicals [33,34], which react with DNA, thus demonstrating its genotoxic effect. Although the mechanisms underlying the protective effect against DNA damage and ACF induction are not clearly understood, the putative antioxidant activity of the *C. langsdorffii* extract might explain its inhibitory action at least in part.

Phenolic compounds display antioxidant activity as a result of their capacity to scavenge free radicals [35]. The most important natural phenolics are flavonoids, which present a broad spectrum of chemical and biological activities, including antioxidant and free radical scavenging properties. Flavonoids act as antioxidants, scavengers of a wide range of reactive oxygen species, and inhibitors of lipid peroxidation [36].

The hydroalcoholic extract of *C. langsdorffii* leaves contains the flavonoids quercetin-3-O-*α*-L-rhamnopyranoside and kaempferol-3-O-*α*-L-rhamnopyranoside as major compounds; these flavonoids constitute about 10% of the crude extract (Figure 3). The high water solubility of this extract facilitates its dissolution in the gastrointestinal tract, enhancing its antioxidant potential. Studies using a model system for food products have demonstrated that flavonoids such as quercetin, rhamnetin, kaempferol, rutin, and quercetrin are one of the most effective antioxidants [37].

Serpeloni et al. [38] stated that studying the chemical constituents of plants and their mechanisms of action is a major challenge: plant species contain various compounds, and their extracts can produce biological effects that may arise from the combined effects of the individual compounds. Phytochemical screening considers that every compound, regardless of their proportion in the plant, known or not, may be an active ingredient [39]. Studies suggest that combinations of phenolics naturally present in fruits and vegetables as a whole inhibit cancer cell growth more effectively than the individual compounds [40].

Flavonoids and other phenolic compounds might exert direct protective effects on the gastrointestinal tract, by scavenging reactive species and/or preventing their formation. Polyphenols can inhibit hemeprotein-induced peroxidation in the stomach and decrease DNA base deamination or nitrosamine formation by HNO_2_-derived reactive nitrogen species [41].

## Conclusion

Our results indicate that the hydroalcoholic extract of *C. langsdorffii* leaves acts as a chemopreventive agent in the rat-DMH colon cancer model. *C. langsdorffii* extract supplementation inhibited DMH-induced DNA damage and ACF formation. Although the mechanism through which the *C. langsdorffii* extract acts in this colon cancer model is unclear, this extract is a promising candidate for the prevention of various types of cancer. Further investigations using *in vitro* assays to elucidate the mechanisms of action of the *C. langsdorffii* extract are desirable before it is used as a chemopreventive agent in humans.

## Competing interest

The authors declare that they have no competing interests.

## Authors’ contributions

JJI, and E carried out the comet and ACF assays. R and J carried out HPLC analysis of the crude hydroalcoholic extract of *Copaifera langsdorffii*. D conceived the study, participated in its design, coordinated, and helped draft the manuscript. All the authors read and approved the final manuscript.

## Pre-publication history

The pre-publication history for this paper can be accessed here:

http://www.biomedcentral.com/1472-6882/13/3/prepub
